# Retreatment with talquetamab and pomalidomide in triple‐class–BCMA–GPRC5D refractory myeloma

**DOI:** 10.1111/bjh.70540

**Published:** 2026-05-10

**Authors:** Kian J. Rahbari, Gliceida M. Galarza Fortuna, Bhagirathbhai Dholaria, Douglas W. Sborov, Muhamed Baljevic

**Affiliations:** ^1^ Department of Internal Medicine Vanderbilt University Medical Center Nashville Tennessee USA; ^2^ Huntsman Cancer Institute, University of Utah Salt Lake City Utah USA; ^3^ Vanderbilt‐Ingram Cancer Center Vanderbilt University Medical Center Nashville Tennessee USA

**Keywords:** BCMA, GPRC5D, immunotherapy, multi‐class refractory multiple myeloma, multiple myeloma, pomalidomide, talquetamab, T‐cell redirecting therapy, triple‐class refractory multiple myeloma

To the Editor,

Despite the introduction of effective T‐cell redirecting (TCR) therapies against B‐cell maturation antigen (BCMA) and G protein‐coupled receptor, class C, group 5, member D (GPRC5D) in the treatment of relapsed/refractory multiple myeloma (RRMM), patients with multiclass refractory disease continue to have dismal outcomes, as demonstrated in the LocoMMotion study, which observed an expected median progression‐free survival (PFS) in triple‐class‐exposed RRMM of 4.6 months, and median overall survival (OS) of approximately 1 year.^1^ Furthermore, multi‐class refractory patients who have progressed after BCMA‐ and/or GPRC5D‐based therapies represent a group of highly challenging patients with uncertain treatment options.^2^ These patients underscore the critical need not only for treatments with novel mechanisms but also innovations in sequencing and combinations of the current armamentarium, which can leverage novel therapeutic synergies. Herein, we present three cases of multi‐class refractory multiple myeloma (MM) patients achieving rapid, deep responses after pomalidomide was reintroduced in combination with every 2‐ to 3‐week (Q2W or Q3W) talquetamab (Talq/Pom) despite previously being refractory to both drugs individually.

The patients in our report were triple‐class/BCMA/GPRC5D refractory (2 were also XPO‐1‐refractory) with multiple high‐risk features. All patients were refractory to pomalidomide from their second‐line treatment. The first patient achieved a stringent complete response (sCR), the second patient achieved a complete response (CR) and the third patient achieved an sCR, the first two after only a few cycles of therapy (Figure 1). All remain on Talq/Pom at 11, 11 and 8 months, respectively, with adequate tolerance (Table 1).

**FIGURE 1:**
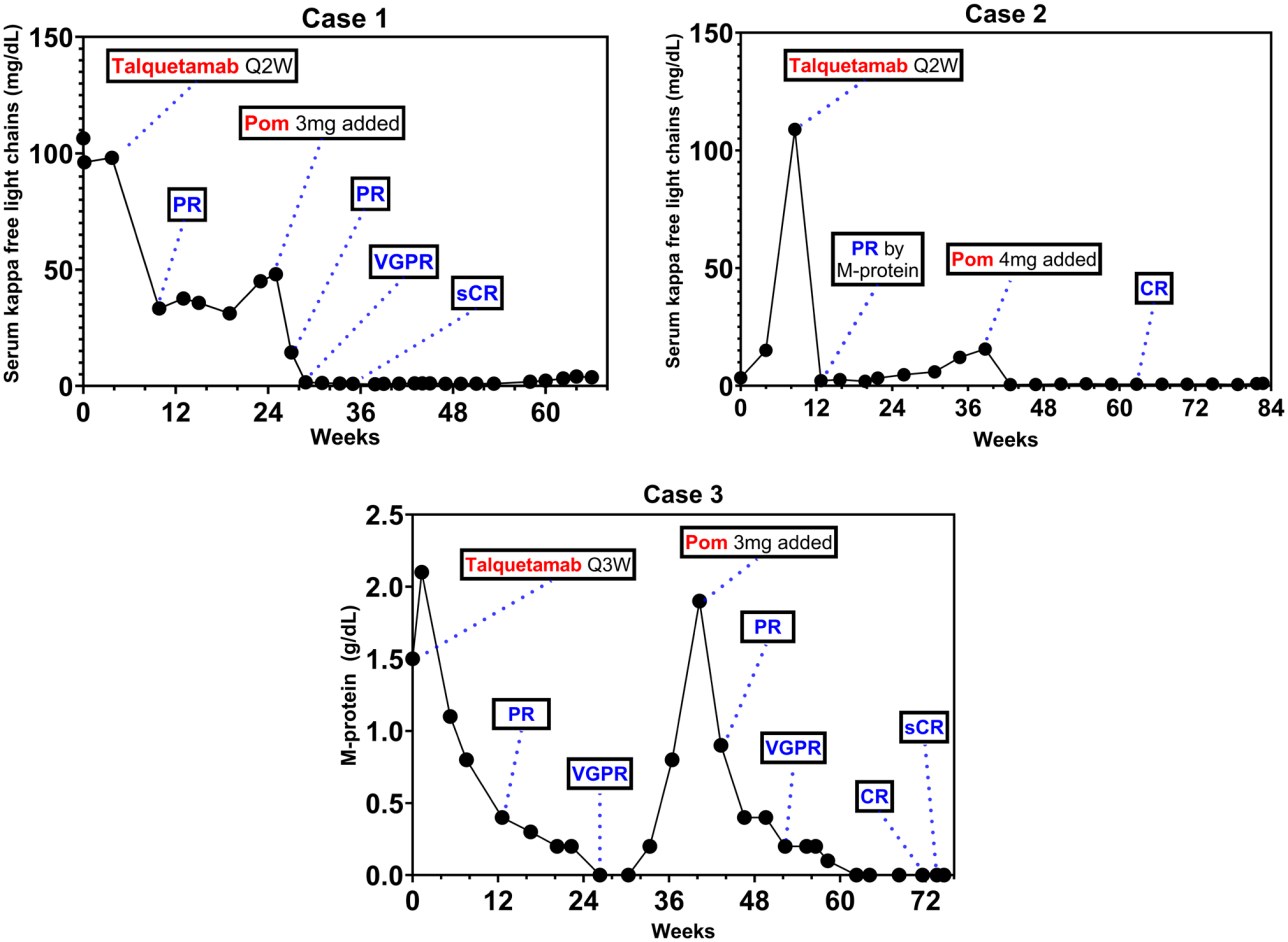
Timeline of patient cases. A graphical summary of the levels of each patient's active secretory marker over time, from the introduction of talquetamab to the present, annotated when pomalidomide was added to therapy and when they met various clinical response criteria. CR, complete response; M‐protein, monoclonal protein; Pom, pomalidomide; PR, partial response; Q2W, every two 2 weeks; Q3W, every three 3 weeks; sCR, stringent complete response; VGPR, very good partial response.

**TABLE 1:** Summary of treatment regimens.

Myeloma regimen	Duration (months)	Best response
1A: Case 1		
(1) VRd, HD‐ASCT, Rev maintenance	24 months	CR
(2) DPd	11 months	VGPR
(3) Anti‐BCMA allo CAR‐T on study	1 month	PD
(4) SKd	4 months	SD
(5) Etentamig on study	2 months	PD
(6) Talquetamab	4 months	PR
(7) Talquetamab + Pomalidomide	11 months →	**sCR**
1B: Case 2		
(1) VRd, HD‐ASCT	5 months	CR
(2) KRd maintenance, Rev maintenance	71 months	sCR
(3) DPd	12 months	SD
(4) KCd	23 months	SD
(5) Teclistamab	9 months	PR
(6) Talquetamab	7 months	PR
(7) Talquetamab + Pomalidomide	11 months →	**CR**
1C: Case 3		
(1) DKRd	4 months	VGPR
(2) SPd	4 months	PD
(3) Anti‐BCMA allo CAR‐T on study	7 months	VGPR
(4) Talquetamab	10 months	VGPR
(5) Talquetamab + Pomalidomide	8 months →	**sCR**

*Note*: A full list of each patient's therapies for multiple myeloma from diagnosis to the present, including the duration of and best clinical response to each therapy.

Abbreviations: ⟶, ongoing response; allo, allogeneic; BCMA, B‐cell maturation antigen; CAR‐T, chimeric antigen receptor T cell; CR, complete response; DKRd, daratumumab, carfilzomib, lenalidomide, dexamethasone; DPd, daratumumab, pomalidomide, dexamethasone; HD‐ASCT, high‐dose melphalan with autologous stem cell transplant; KCd, carfilzomib, cyclophosphamide, dexamethasone; KRd, carfilzomib, lenalidomide, dexamethasone; PD, progressive disease; PR, partial response; Rev, lenalidomide; SD, stable disease; SKd, selinexor, carfilzomib, dexamethasone; SPd, selinexor, pomalidomide, dexamethasone; VRd, bortezomib, lenalidomide, dexamethasone.

We wish to highlight an exceptional response of late‐line pomalidomide when reintroduced to talquetamab, in three patients who were refractory to both agents. While the Talq/Pom combination is still under active investigation in the pomalidomide non‐refractory setting, a curious finding of deep and rapid responses highlights the potential value of this combination in the absence of other treatment options. It further underscores the need for a thoughtful approach to optimal therapeutic sequencing and combinations in the rapidly expanding T‐cell redirection space.

The first patient is a 68‐year‐old woman with revised international staging system (R‐ISS) stage 2, 1q21.3 gain kappa light chain—predominant MM, who was started on talquetamab as her sixth line of treatment. Her prior therapies are summarized in Table 1A and included an autologous stem cell transplant (AHSCT), two immunomodulator (IMiD) therapies including pomalidomide, two proteasome inhibitors, an anti‐CD38 antibody and an anti‐BCMA chimeric antigen receptor (CAR) T‐cell product. She achieved a partial response (PR) to Q2W talquetamab but progressed after 4 months by International Myeloma Working Group (IMWG) light chain criteria, with serum kappa light chains increasing to 48 mg/dL from a nadir of 33 mg/dL. Pomalidomide was reintroduced at 3 mg daily for 21 of each 28‐day cycle as a temporizing measure while clinical trials were considered. However, she experienced a dramatic response, achieving a stringent complete response (sCR) after 8 weeks on therapy (Figure 1). She has tolerated therapy well, with grade 2 neutropenia and mild nail changes without infection or cytokine release syndrome (CRS). She maintains her response after over 11 months.

The second patient, a 55‐year‐old man with *TP53*‐mutated, quad‐class refractory, IgG kappa MM with extramedullary disease, progressed through teclistamab and was started on Q2W talquetamab in the sixth‐line setting (Table 1B). He achieved a PR with a 50% reduction in serum monoclonal protein (M‐protein) after 1 month, and though his M‐protein remained stable, his light chains consistently trended upwards: by the 7th month, this trend accelerated, with doubling of kappa light chain up to 15 mg/dL over the preceding 2 months from a nadir of 1 mg/dL, demonstrating light chain escape while M‐protein remained without major changes. Given the patient's high‐risk disease, this biochemical relapse/conversion was designated as warranting change in treatment, and pomalidomide 4 mg 21/28 was added. His light chains normalized within 1 month, and after 11 months, he is in confirmed complete response (CR). He experienced fevers requiring pomalidomide dose reduction to 2 mg but otherwise has had only mild itching and dysgeusia.

The third patient is a 71‐year‐old woman with triple‐class refractory, 1q21.3 gain R‐ISS stage 2, transplant‐ineligible, IgG lambda MM with extramedullary disease, who started Q2W talquetamab in the fourth‐line setting (Table 1C) after previously progressing through an anti‐BCMA CAR‐T product. She had an excellent initial response to Q2W talquetamab but began to rapidly progress, with M‐protein increasing from undetectable to 1.9 mg/dL, so pomalidomide 3 mg 21/28 was added to Q3W talquetamab due to cytopenias. After 1 month, she achieved a PR and shortly after was found to be in a very good partial response (VGPR) by M‐protein criteria. Her response continued to deepen, achieving a VGPR after 3 months and a CR after 7 months, with sCR confirmed after 8 months. She now maintains her sCR at 8 months with therapy complicated only by persistent grade 2–3 cytopenias not requiring transfusion.

TCR therapies have transformed the prognosis of RRMM, but further insights into disease biology are needed to optimize their use. We wish to highlight an exceptional therapeutic response of late‐line Talq/Pom in three multi‐class refractory patients who were previously refractory to both agents.

Resistance to TCR therapies is mediated through T‐cell dysfunction, antigenic escape and tumour microenvironmental factors such as local hypoxia or the presence of inhibitory leucocytes.^3^ Antigenic escape with loss of BCMA or GPRC5D surface expression is prevalent among relapsed cases and is thus considered to be the primary driver,^4^ but the relative impact of each of these mechanisms, as well as the sequence of events, is unknown and likely unique to each individual. In preclinical models, pre‐existing T‐cell fitness has been shown to be critical in predicting response to TCR therapy,^5^ and combining a TCR therapy with an IMiD has been shown to improve T‐cell fitness by increasing CD8/CD4 ratios, increasing differentiation to stem cell‐like memory T cells and decreasing T‐regulatory cells—ultimately improving responses in a mouse model.^6^ Similar effects have been demonstrated by exportin‐1 inhibitors and Cereblon E3 Ligase Modulatory Drugs (CELMoDs) which may overcome T‐cell exhaustion induced by TCR therapies.^7^, ^8^, ^9^

Currently, there are no established means to overcome TCR therapy resistance and permit retreatment with the same agent. Given that all three patients in the present report were definitionally refractory to pomalidomide, their response to Talq/Pom suggests a synergy between the two drugs which allowed them to overcome an emerging resistance to talquetamab. These findings are novel, and though the combination of Talq/Pom has been studied in the MonumenTAL‐2 trial, only 5% of patients were pomalidomide refractory and none had prior GPRC5D exposure.^10^ The TRIMM‐2 study reported on a cohort with Talq/Pom plus daratumumab, of whom 70% were pomalidomide refractory, but this trial also excluded prior GPRC5D exposure.^11^ Talq/Pom is under active investigation in patients without prior pomalidomide exposure in MonumenTAL‐3.

While these striking clinical observations offer no direct insights into mechanism, they demonstrate a potentially valuable clinical option for multi‐class refractory patients with no established standards of care, and they raise the question of whether modulating T‐cell fitness may overcome resistance to TCR therapies. The intriguing findings in these patients highlight the potential value of Talq/Pom after exhausting standard of care options while other treatments such as clinical trials are being explored—particularly as a salvage option after progressing through TCR monotherapy—and they underscore the need for thoughtful clinical trials to tackle the essential question of optimal therapeutic pairing and sequencing in the rapidly expanding TCR space.

## AUTHOR CONTRIBUTIONS

All authors were directly involved in writing and revising the manuscript. K.J.R produced the figures and table, which were reviewed by all authors.

## FUNDING INFORMATION

This work received no funding.

## CONFLICT OF INTEREST STATEMENT

Dr. Rahbari and Dr. Galarza Fortuna declare no competing interests. Dr. Dholaria has received compensation for consulting or advisory roles for MJH BioScience, Arivan Research, Janssen, ADC therapeutics, Gilead, GSK, Caribou, Roche, Autolus, Poseida, Pierre Fabre, AstraZeneca, Arima Genomics as well as institutional research funding from Janssen, Angiocrine, Pfizer, Poseida, MEI, Orcabio, Wugen, Allovir, Adicet, BMS, Molecular template, Atara, Merck. Dr. Sborov has received compensation for consulting or advisory roles for Abbvie, Arcellx, AstraZeneca, BiolineRx, Bristol‐Myers Squibb/Celgene, Genentech, GlaxoSmithKline, Janssen, Opna Bio, Pfizer and Sanofi as well as research funding from Pfizer. Dr. Baljevic has received compensation for consultancies with Pfizer, AbbVie, J&J; for serving on advisory boards for BMS/Celgene, Janssen Biotech, J&J, Sanofi‐Genzyme, Pfizer, Prothena, AbbVie; and as part of IRCs for Parexel International.

## ETHICS STATEMENT

The current work was exempted from requiring Ethics approval and consent to participate based on institutional guidelines.

## PATIENT CONSENT STATEMENT

The patients in this report provided consent to publish these deidentified findings.

## Data Availability

The data that support the findings of this study are available on request from the corresponding author. The data are not publicly available due to privacy or ethical restrictions.
